# Omega-3 Fatty Acids in Arterial Hypertension: Is There Any Good News?

**DOI:** 10.3390/ijms24119520

**Published:** 2023-05-30

**Authors:** Gabriele Brosolo, Andrea Da Porto, Stefano Marcante, Alessandro Picci, Filippo Capilupi, Patrizio Capilupi, Nicole Bertin, Cinzia Vivarelli, Luca Bulfone, Antonio Vacca, Cristiana Catena, Leonardo A. Sechi

**Affiliations:** 1 Department of Medicine, University of Udine, 33100 Udine, Italy; andrea.daporto@uniud.it (A.D.P.); stefanomarcante@outlook.it (S.M.); aless.picci@gmail.com (A.P.); filippocapilupi@gmail.com (F.C.); patrizio.capilupi@gmail.com (P.C.); nicole.bertin@uniud.it (N.B.); cinzia.vivarelli@asufc.sanita.fvg.it (C.V.); luca.bulfone1@gmail.com (L.B.); antonio.vacca94@gmail.com (A.V.); cristiana.catena@uniud.it (C.C.); 2 European Hypertension Excellence Center, Clinica Medica, University of Udine, 33100 Udine, Italy; 3 Diabetes and Metabolism Unit, Clinica Medica, University of Udine, 33100 Udine, Italy; 4 Thrombosis and Hemostasis Unit, Clinica Medica, University of Udine, 33100 Udine, Italy

**Keywords:** omega-3 polyunsaturated fatty acids (ω-3 PUFAs), hypertension, linoleic acid (LA), alpha-linoleic acid (ALA), eicosapentaenoic acid (EPA), docosahexaenoic acid (DHA), oxylipins, endothelial dysfunction, arterial stiffness, primary prevention, secondary prevention, residual cardiovascular risk

## Abstract

Omega-3 polyunsaturated fatty acids (ω-3 PUFAs), including alpha-linolenic acid (ALA) and its derivatives eicosapentaenoic acid (EPA) and docosahexaenoic acid (DHA), are “essential” fatty acids mainly obtained from diet sources comprising plant oils, marine blue fish, and commercially available fish oil supplements. Many epidemiological and retrospective studies suggested that ω-3 PUFA consumption decreases the risk of cardiovascular disease, but results of early intervention trials have not consistently confirmed this effect. In recent years, some large-scale randomized controlled trials have shed new light on the potential role of ω-3 PUFAs, particularly high-dose EPA-only formulations, in cardiovascular prevention, making them an attractive tool for the treatment of “residual” cardiovascular risk. ω-3 PUFAs' beneficial effects on cardiovascular outcomes go far beyond the reduction in triglyceride levels and are thought to be mediated by their broadly documented “pleiotropic” actions, most of which are directed to vascular protection. A considerable number of clinical studies and meta-analyses suggest the beneficial effects of ω-3 PUFAs in the regulation of blood pressure in hypertensive and normotensive subjects. These effects occur mostly through regulation of the vascular tone that could be mediated by both endothelium-dependent and independent mechanisms. In this narrative review, we summarize the results of both experimental and clinical studies that evaluated the effect of ω-3 PUFAs on blood pressure, highlighting the mechanisms of their action on the vascular system and their possible impact on hypertension, hypertension-related vascular damage, and, ultimately, cardiovascular outcomes.

## 1. Introduction

Arterial hypertension is the most frequent chronic disease worldwide [1,2], with a prevalence that reaches 35% of the adult population accounting for 9.4 million yearly deaths [3]. Hypertension is a major modifiable cardiovascular risk factor, increasing the risk of cardiovascular death and morbidity related to chronic invalidating disorders, including coronary, cerebrovascular, and peripheral vascular disease, heart failure, and chronic kidney disease [4]. Primary hypertension is a complex pathophysiological condition that is characterized at the peripheral vascular level by an imbalance between vasoconstriction and vasodilatation [5]. This imbalance arises from an intricate interplay of genetic and environmental factors that include lifestyle and dietary habits [6]. In this regard and due to their proven ability to reduce blood pressure (BP), lifestyle changes are highly recommended in all patients as the first step to treat hypertension [1,2]. Among lifestyle recommendations, dietary interventions play an essential role to the extent that caloric restriction contributes to weight loss and supplementation with specific food components affects BP regulation. 

In the last three decades, omega-3 polyunsaturated fatty acids (ω-3 PUFAs) have gained great interest within the research community, since seminal studies [7,8,9,10] reported a surprisingly low incidence of cardiovascular disease in populations, such as Eskimos and Alaskan Natives, traditionally eating high amounts of ω-3 PUFA-rich fatty fish. Later, epidemiological, observational, interventional studies, comprehensive reviews, and meta-analyses were performed with the aim to define the true potential of ω-3 PUFAs for cardiovascular prevention. Moreover, in vitro and in vivo animal and human studies have paved the way for a better understanding of the cellular and molecular mechanisms of ω-3 PUFA-mediated vascular protection. These studies have clearly demonstrated that ω-3 PUFAs possess antioxidant, anti-inflammatory, antithrombotic, and endothelium protective properties [11]. In addition, dietary supplementation with high doses of ω-3 PUFAs modifies plasma lipids concentrations decreasing serum triglyceride and increasing HDL-cholesterol levels [12,13,14,15]. Evidence suggests that triglyceride-rich lipoproteins contribute to the “residual lipoprotein attributable risk” that was reported in high-risk patients treated with high-dose statins, despite reaching very low LDL-cholesterol levels [16,17,18,19]. Despite inconsistent results of early randomized controlled trials with triglyceride-lowering drugs, subgroup analyses of more recent studies [20,21,22] and the results of the REDUCE-IT (Reduction of Cardiovascular Events with Icosapent Ethyl Intervention Trial) [23] showed a significant cardiovascular benefit of triglyceride-lowering, thus expanding the potential role of ω-3 PUFAs for clinical practice. As a result, national and international guidelines for cardiovascular prevention currently recommend regular consumption of ω-3 PUFAs as part of a healthy diet [24,25,26,27]. Moreover, ω-3 PUFA-deficient diets have been classified as the sixth most relevant dietary risk factor, accounting for 1.5 million deaths and 33 million disability-adjusted life years worldwide [28].

This narrative review aims to provide an update on the current views of the relevance of ω-3 PUFAs in arterial hypertension. For the purpose of this review, we systematically searched the medical literature in the English language using the PubMed MeSH and the terms «omega 3», «polyunsaturated fatty acids», «fish fat», «blood pressure», and «arterial hypertension» for extraction. Publications considered were full-text articles with original human or experimental data for the effect of ω-3 PUFAs on BP and its regulatory mechanisms and meta-analyses and reviews on this same subject. G.B. retrieved the articles that were reviewed and discussed with C.C. and L.A.S. for subsequent article selection. Article selection was performed according to the quality of evidence of studies that was estimated following the Grading of Recommendations, Assessment, Development, and Evaluations (GRADE) criteria that are based on the study design, dimension, consistency, and magnitude and dose-dependency of effect [29]. Only studies rated with moderate-to-high GRADE certainty ratings were considered.

## 2. Biochemistry and Cellular Mechanisms of Polyunsaturated Fatty Acids (PUFAs)

### 2.1. General Biochemistry and Biologic Sources of PUFAs

**Figure 1:**
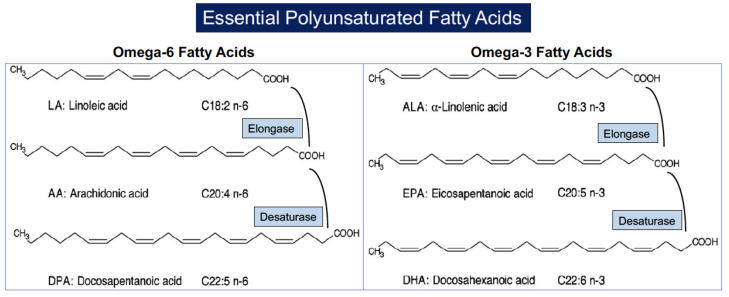
Structure of polyunsaturated fatty acids and their formation from parental molecules.

The nomenclature of PUFAs is based on the number of carbons in the molecule (*C*) and the position of the first double bond relative to the terminal methyl carbon. Omega (ω) indicates the last methyl carbon as opposed to the carboxyl group of the acyl chain, and *-6* or *-3* indicates the position of the first double bond from the last methyl group [30]. PUFAs are categorized into essential and nonessential according to the ability of the human body to synthesize them de novo. Nonessential PUFAs are those of the ω-7 and ω-9 families since they can be synthesized directly from endogenous saturated fatty acids. Essential PUFAs include the ω-6 and ω-3 families, which cannot be synthesized de novo because mammals lack enzymes to build up double bonds in the fatty acid chain and must necessarily be obtained from the diet. The two parents essential PUFAs are linoleic acid (C18:2, LA) that is particularly abundant in vegetable oils derived from soybean, corn, sunflowers, and rapeseed and alpha-linoleic acid (C18:3, ALA) that is predominantly found in flaxseed, soybean, canola oils, pumpkin seeds and pumpkin seed oil, perilla seed oil, tofu, walnuts and walnut oil, camelina, hempseed, and some algae. Linoleic acid is the precursor of ω-6 PUFAs and is converted into arachidonic acid (C20:4, AA) through the action of elongases (which add carbons to the hydrocarbon chain of the fatty acid) and desaturases (which replace single bonds with double bonds). Alpha-linoleic acid (C18:3, ALA) is the precursor of long-chain ω-3 PUFAs through elongation and desaturation of its acyl chain, leading to the formation of eicosapentaenoic acid (C20:5, EPA) which in turn is converted into docosahexaenoic acid (C22:6, DHA) [31,32] (Figure 1).

Although humans can endogenously synthesize long-chain ω-3 PUFAs (EPA, DHA) by elongation and desaturation of dietary ALA, it must be emphasized that these reactions in humans are slow and inefficient, so that the production rate of these compounds is very low [33]. The rate of conversion of ALA to EPA and DHA was estimated to be 20% and 10%, respectively, in women, with apparently lower values in men (8% and 10% correspondingly) [34,35,36]. The main source of EPA and DHA is indeed exogenous, mainly derived from fatty fish and seafood (mackerel, salmon, trout, seabass, oysters, sardines, and shrimp). A further element of complexity is represented by the fact that ω-3 PUFAs and ω-6 PUFAs both compete for the same enzymatic pathway involving elongases and desaturases, leading to the transformation of precursors ALA and LA into EPA/DHA and AA, respectively [37,38]. Although ALA is a more affine substrate for desaturases and elongases, some experimental evidence has demonstrated a slower enzymatic metabolism of ω-3 PUFAs as compared to ω-6 PUFAs [39]. Indeed, adequate intake of both ALA and LA is of paramount importance for human health, and the relative dietary proportions of precursor fatty acids (LA, ALA) determine the net rate of conversion to their respective long-chain derivatives [40,41]. Currently, western diets are abundant in ω-6 PUFAs, being found in meats and poultry alongside deep-fried foodstuffs, a characteristic that favors high LA intake, and relatively deficient in ω-3 PUFAs, with ratios of ω-6 PUFAs to ω-3 PUFAs as high as 15:1 to 16.7:1 [42]. Moreover, research suggests that most of the world's population including those who consume fish regularly have a low omega-3 index (*O3i*). This is defined as the ratio of EPA and DHA to total fatty acids in erythrocyte membranes and as a marker of overall ω-3 PUFA status and values below 8% [43,44] have been previously validated as a risk factor for cardiovascular disease [45].

### 2.2. PUFAs and Cellular Membranes 

As structural components of membrane phospholipids, PUFAs influence membrane properties and modulate cellular function. Fatty acids are quickly incorporated in phospholipids of plasma, platelets, neutrophils, and red blood cells, whereas enrichment of other tissues takes a longer time. EPA and DHA interact differently with cellular membranes [46,47,48]. Preclinical data show that EPA has a more stable interaction with surrounding saturated fatty acids, contributing to membrane stability and inhibiting lipid oxidation and cholesterol domain formation. DHA is in a curved shape causing conformational changes that increase membrane fluidity and form cholesterol domains with reduced antioxidant activity. These peculiarities may at least in part account for the differences in their clinical effects, with EPA seemingly more efficient in the mitigation of the atherosclerotic process. ω-3 PUFAs accumulate preferentially in the cerebral cortex, retina, testes, muscle, and liver, while ῳ-6 PUFAs are ubiquitous in all tissues. ω-3 PUFAs are incorporated into membrane phospholipids, where they usually represent less than 10% of the total amount of fatty acids (in a typical western diet: 10–20% AA, 0.5–1% EPA, and 2–4% DHA). Of note, the dietary intake of ω-3 PUFAs can modify the composition of cell membranes in a relatively short time (from days to weeks) [49,50,51].

Plasma membranes consist of a mosaic of functional microdomains that facilitate interactions between resident proteins and lipids [52,53]. The lipid content of cell membranes affects the function of cells and intracellular organelles. Incorporation of ω-3 PUFAs into these membranes modifies membrane fluidity and biophysics, size, and composition of these microdomains, namely, lipid rafts and caveolae, that modulate protein function and signaling events. Lipid rafts are small heterogeneous membrane microdomains rich in cholesterol, sphingolipids, and saturated acyl chain [53,54] that influence membrane fluidity, protein–protein interaction, ion channel kinetics, signaling processes, and membrane protein and receptor trafficking. Caveolae represent a specific subtype of lipid raft macrodomain that forms flask-shaped membrane invaginations rich in proteins that play a role in endocytosis and signal transduction, including the structural protein caveolin-1 and many signal transduction proteins [55]. ω-3 PUFAs have been shown to increase the size and to change the content of cholesterol and sphingolipids of lipid rafts because of their low polar affinity with these types of lipids. These changes by modulating signaling events such as activation of G-protein, endothelial nitric oxide synthase (eNOS), tumor necrosis factor alpha (TNF-alpha), adhesion molecules, and Toll-like receptors (TLR) can contribute to the anti-inflammatory and antiatherosclerotic properties of ω-3 PUFAs [56,57,58]. For example, enrichment of cellular membranes with ω-3 PUFAs disrupts dimerization and recruitment of toll-like receptor-4, which might contribute to anti-inflammatory effects by down-regulation of nuclear factor-kappa B (NF-ⱪB) activation. On the other hand, the incorporation of ω-3 PUFAs into lipid membranes might modulate a variety of ion channels [59]. These include certain voltage-gated (K_v_) and inwardly rectifying (K_ir_) K^+^ channels, voltage-gated (Na_v_) and epithelial (ENaC) Na^+^ channels, L-type Ca^2+^ channels, hyperpolarization-activated cyclic nucleotide-gated (HCN) channels, transient receptor potential (TRP) channels, various connexins, chloride channels, and P2X receptors [59].

### 2.3. PUFA and Intracellular Signaling 

Modulation of physicochemical properties of cellular and organelle membranes is not the only mechanism through which ω-3 PUFAs exert their physiological effects. ω-3 PUFAs directly interact with membrane channels and proteins. For example, direct modulation of ion channels or G-protein-coupled receptor 120 (GPR 120) might contribute to antiarrhythmic or anti-inflammatory effects, respectively [60,61,62]. GPR 120, highly expressed in human adipocytes and macrophages, has been identified in recent years as the receptor of ω-3 PUFAs [63,64] and has been consequently renamed “Free fatty acid receptor 4” (FFAR4). Binding and activation of FFAR4 by ω-3 PUFAs in macrophages and Kupffer cells trigger a downstream signaling cascade leading to the assembly of ῳ-3PUFA/FFAR4/β-arrestin-2 complex, which in turn dissociates the TAK1/TAB1 heterodimer by binding to and inactivating the TAB1 subunit, consequently reducing NF-κB-mediated cyclooxygenase expression and inflammation [62]. Interestingly, in a study of rodents and lipopolysaccharide-primed bone-marrow-derived macrophages, the ῳ-3PUFA/FFAR4/β-arrestin-2 complex has also demonstrated to inhibit NOD-like receptor protein 3 (NLRP3) inflammasome-dependent inflammation [65]. NLRP3 inflammasome has been identified in the last decade as a functional bridge between inflammation and atherosclerosis [66], and its downregulation results in decreased levels of interleukin-1β and decreased production of interleukin-6 release by macrophages and C-reactive protein by hepatocytes. Moreover, ω-3 PUFAs directly regulate gene expression via nuclear receptors and transcription factors, being the natural ligands of many key nuclear receptors in multiple tissues, including peroxisome proliferator-activated receptors (PPAR-alpha, -beta, -delta, and -gamma), hepatic nuclear factors (HNF-4; -alpha and -gamma), retinoid X receptors (RXR), and liver X receptors (alpha and beta) [67,68,69,70,71,72]. ω-3 PUFAs are transported into the nucleus by cytoplasmic lipid-binding proteins. Sterol regulatory element binding protein-1c (SREBP-1c) is an example of a transcription factor whose function is altered by ω-3 PUFAs, contributing to their effects on lipid metabolism and inflammatory pathways [73,74].

### 2.4. PUFAs and Its Metabolites: Role of Oxylipins

A further mechanism of action of ω-3 PUFAs directly involves their metabolites (ω-3 Oxylipins) that in recent years have gathered great research interest. After being released from phospholipids by cytosolic phospholipase A2 (cPLA2), both ω-6 PUFAs and ω-3 PUFAs are oxygenated by different enzymes, such as cyclooxygenase (COX), lipoxygenase (LOX), and cytochrome P450 (CYP) enzymes. This leads to the synthesis of a broad variety of bioactive lipid compounds, many of which take part in the regulation of vascular function and exert potent anti-inflammatory effects [75,76]. “Classic oxylipins” are eicosanoids (including prostaglandins, thromboxanes, and leukotrienes) derived from both ῳ-6 PUFAs and ω-3 PUFAs. ω-6 PUFA AA is the precursor of two types of prostaglandins, thromboxanes, and four types of leukotrienes with strong pro-inflammatory, pro-thrombotic, and vasoconstrictive properties [77,78]. EPA is the precursor of three types of prostaglandins and five types of leukotrienes [38] that are 10- to 100-fold less biologically active than their counterparts that are derived from AA and are antagonistic with their effects on vascular tone, platelet aggregation, and inflammation [77]. In this context, when balancing of enzymatic conversions favors 3-series thromboxanes (TXA3 vs. TXA2) and prostacyclines (PGI3 vs. PGI2), the effects of PGI prevail because of the relative power of these molecules. In vitro and in vivo experiments show that AA metabolizing P450-enzymes can use EPA and DHA as alternative substrates. Therefore, EPA/DHA supplementation shifts the P450-eicosanoid profile to EPA- and DHA-derived epoxy- and hydroxy-metabolites (17,18-epoxy-EPA and 19,20-epoxy-DHA) that are commonly identified as CYP eicosanoids [79]. These eicosanoids show protective vasoactive actions and antiarrhythmic properties in cardiomyocytes and are linked to the development of hypertension, myocardial infarction, pathological cardiac hypertrophy, stroke, kidney injury, and other inflammatory disorders [79,80]. Besides “classic oxylipins”, ω-3 PUFAs can generate some other oxylipins, designated “specialized pro-resolving mediators” (SPM), that participate in the resolution of inflammation and exert protective and beneficial effects on a variety of inflammatory diseases [81,82]. These compounds include resolvins, protectins, and maresins. EPA generates E-series resolvins (RvE1, RvE2, and RvE3) through the action of COX, while D-series resolvins (RvD1-D6), protectins, and maresins (including MaR1 and MaR2) are derived from DHA, through the actions of LOX [81]. Moreover, some ῳ-3 oxylipins, including protectin DX, maresin 1, and resolvin D1, display antioxidant capacity by regulating the expression of antioxidant proteins including catalase, superoxide dismutase, and glutathione peroxidase activity and attenuating lipid peroxidation and O_2_-generation [83,84].

**Figure 2:**
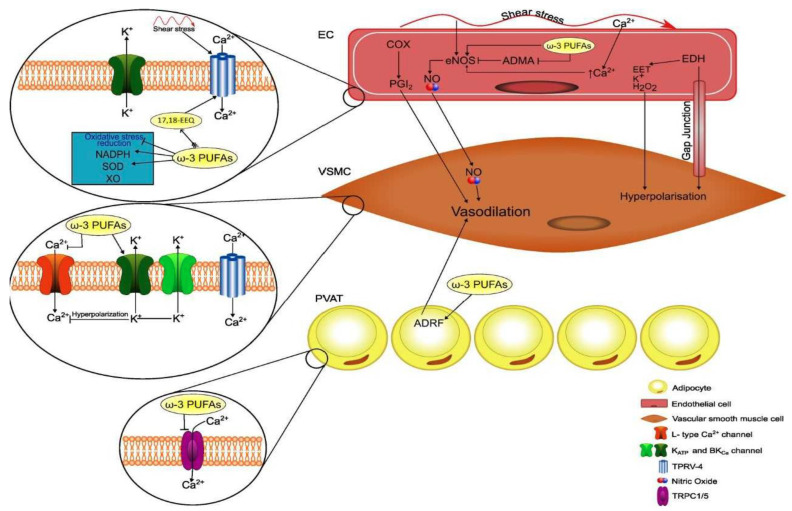
Schematic representation of actions of omega-3 polyunsaturated fatty acids (ω-3 PUFAs) on cell membranes and intracellular signaling and their relevance for perivascular adipocytes and vascular cell functions. EEQ, epoxyeicosatetraenoic acid; NADPH, nicotinamide adenine dinucleotide; SOD, superoxide dismutase; XO, xanthine oxidase; PVAT, perivascular adipose tissue; ADRF, adventitium-derived relaxing factor; COX, cyclooxygenase; PGI2, prostacyclin; NO, nitric oxide; eNOS, endothelial nitric oxide synthase; ADMA, asymmetric dimethylarginine; EET, epoxyeicosatrienoic acid; endothelium-derived hyperpolarizing factor; BKCa, large-conductance voltage- and calcium-activated potassium channel; TRPV-4, transient receptor potential vanilloid-4; TRPC1/5, transient receptor potential cation.

In summary, the incorporation of ω-3 PUFAs in membrane phospholipids improves membrane fluidity and biophysical properties by changing lipid rafts and caveolae characteristics, thereby leading to modulation of protein–protein interaction and ion channel kinetics. These membrane changes involve also intracellular organelles and trigger a multiplicity of intracellular signaling mechanisms that can contribute to the antiatherosclerotic properties of ω-3 PUFAs and to the regulation of peripheral vascular tone (Figure 2).

## 3. ω-3 PUFAs and Blood Pressure

**Figure 3:**
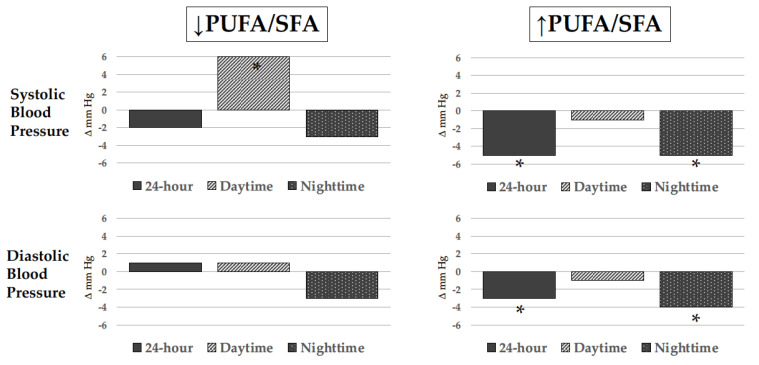
Changes in blood pressure levels as measured by noninvasive ambulatory blood pressure monitoring (ABPM) in hypertensive patients who ate 3 weekly meals of trout rich in polyunsaturated fatty acids for 6 months. Changes are represented according to change (↑ increase; ↓ decrease) in erythrocyte cell membrane polyunsaturated to saturated fatty acid ratio (PUFA/SFA). Only hypertensive patients with an increased PUFA/SFA had significant 24-hour and nighttime blood pressure reduction (adapted from ref. [93]). * *p* < 0.05.

The effect of ω-3 PUFAs on BP has been well characterized in the past three decades across multiple trials, systematic reviews, and meta-analyses, most of which included both hypertensive and normotensive individuals. Overall, meta-analyses have shown that relatively high doses of ω-3 PUFAs, usually more than 3 g/day, lead to small albeit meaningful BP reductions, particularly in subjects with untreated hypertension. The first meta-analysis appeared in 1993 and included 17 controlled clinical trials (6 in untreated hypertensive subjects without any other comorbidity and 11 in normotensives) reporting a reduction in systolic BP (SBP) and diastolic BP (DBP) that was significant only in hypertensives (−5.5 and −3.5 mm Hg, respectively) [85], with a median ω-3 PUFA dose of 5 g/day that was administered for a median of 8 weeks. Another meta-analysis that included 31 controlled clinical trials with 1356 healthy or hypertensive participants confirmed a significant reduction in SBP and DBP only in hypertensive patients (−3.4 and −2 mm Hg, respectively) who took an average ω-3 PUFA dose of 4.8 g/day in the form of fish or fish oil for 3 to 24 weeks [86]. Another meta-analysis included a total of 36 studies, 22 of which had a double-blind design [87]. Fish oil reduced SBP by 2.1 mm Hg and DBP by 1.6 mm Hg with effects that tended to be greater in hypertensive subjects older than 45 years. Effects of ω-3 PUFAs on BP were the object of further subsequent meta-analyses [88,89,90,91,92], all showing a small but significant reduction in BP levels. These results have been recently corroborated by an *umbrella* meta-analysis [93] that included 10 meta-analyses of 131 studies carried out between 1989 and 2021 with ω-3 PUFA supplements across studies of 2.2 to 6 g/day and duration of exposure from 4 to 29 weeks. This meta-analysis has confirmed that ω-3 PUFA decreases SBP (−1.19 mm Hg; 95% CI: −1.76, −0.62, *p* < 0.001) and DBP levels (−0.91 mm Hg, 95% CI: −1.35, −0.47; *p* < 0.001) with effects that were more prominent in older hypertensive subjects who were supplemented with doses greater than 2 g/day for more than 10 weeks. Although results of meta-analyses indicate an effect of ω-3 PUFA supplementation in reducing BP, important variability of findings in individual studies reflecting heterogeneity in the source (fish, fish oil, capsules enriched with ethyl-ester forms of DHA and EPA, powders), the relative content of EPA and DHA, duration of exposure, and differences in terms of comorbidities and additional treatments should not be overlooked [94]. In addition, as for any other dietary intervention study, assessment of compliance to prescription is particularly problematic and only a minority of studies were controlled with measurement of ω-3 PUFA content in cellular membranes. We prospectively followed a group of uncomplicated hypertensive patients who were advised to eat a meal of farmed fish fed with PUFA-enriched chow three times a week for 6 months. Ambulatory BP monitoring and ω-3 PUFA content in red blood cell plasma membranes were evaluated at baseline and at the end of the study. Twenty-four-hour and nighttime BP were significantly reduced only in those patients who had increased membrane ω-3 PUFA content with an effect that was more pronounced in those with lower baseline ω-3 PUFA content [95] (Figure 3).

Although the BP-lowering effect of ω-3 PUFAs was sufficiently well characterized across multiple intervention trials and meta-analyses, observations on the dose–response relationship between ω-3 PUFAs and BP reduction were conflicting. Early meta-analyses suggested a linear relationship [85,87,89], whereas nonlinear relationships were suggested in other meta-analyses that reported either a J-shaped [96] or L-shaped [97] association. In a further and more recent meta-analysis that was specifically designed to analyze the dose–response relationship of ω-3 PUFA intake with BP, Zhang et al. [98] included 71 studies involving 4973 individuals. Results indicated a nonlinear J-shaped association with a greater reduction in BP that was obtained with doses between 2 g/day (SBP, −2.61 mm Hg (95% CI, −3.57 to −1.65); DBP, −1.64 mm Hg (95% CI, −2.29 to −0.99)) and 3 g/day (SBP, −2.61 mm Hg (95% CI, −3.52 to −1.69); DBP, −1.80 mm Hg (95% CI, −2.38 to −1.23)) further providing evidence of a greater effect in untreated hypertensive subjects. This ω-3 PUFA-induced decrease in BP is of clinical significance on a population level, as previous studies have estimated that a 2 mm Hg reduction in SBP reduces stroke mortality by 10% and deaths from ischemic heart disease by 7% [94], leading to 30.045 fewer major cardiovascular events [99]. Thus, current evidence suggests that ω-3 PUFA supplementation might have a potential place in the treatment of low-risk patients with mild grade-1 hypertension in addition to other interventions on lifestyle.

## 4. Antihypertensive Mechanisms of ω-3 PUFAs

BP is the product of cardiac output (heart rate x stroke volume) and peripheral vascular resistance to blood flow. ω-3 PUFAs and the related ῳ-3 oxylipins can influence BP levels by acting both on cardiac hemodynamics and vascular function (Table 1).

### 4.1. ω-3 PUFAs and Cardiac Hemodynamics

A cohort study that included more than 5000 subjects who ate a diet containing a high amount of grilled or baked fish reported that fish consumption three or more times per week was associated with a reduction in heart rate of 3 beats/min [100]. Mild slowing of heart rate was confirmed in a meta-analysis of 30 randomized, controlled studies with ω-3 PUFA supplementation that reported an average 2 beats/min reduction that depended on the duration of exposure but was independent of the ω-3 PUFA dose [101]. Other studies have shown that ω-3 PUFA supplementation decreases heart rate variability and recovery after exercise, both effects that could be mediated by modulation of the vagal tone [102,103]. Other studies in humans and nonhuman primates showed that ω-3 PUFAs increase cardiac stroke volume through the improvement of ventricular diastolic filling [104,105]. In conclusion, cardiac output is poorly affected by ω-3 PUFAs because the increased stroke volume is counterbalanced by a decreased heart rate. Therefore, possible explanations for the BP-lowering effect of ω-3 PUFAs should be nec1essarily sought in the peripheral vasculature.

### 4.2. ω-3 PUFAs and Regulation of Peripheral Vascular Resistance

Clinical studies have repeatedly suggested an association of ω-3 PUFA intake with decreased vascular resistance [106,107] and mechanisms underlying this association were extensively investigated in human and animal studies [108,109]. Although some studies failed to demonstrate improved vascular reactivity with the use of ω-3 PUFAs [110,111,112], a large body of evidence supports the view that these compounds could improve both endothelium-dependent and endothelium-independent peripheral vascular responses.

#### 4.2.1. ω-3 PUFAs and Endothelium-Dependent Regulation of Vascular Tone

Endothelial cells (ECs) play a leading role in the regulation of a variety of vascular functions including vascular tone. ECs synthesize and release several factors, including vasodilators and vasoconstrictors, growth factors and growth inhibitors, pro-thrombotic and antithrombotic factors, and pro-inflammatory and anti-inflammatory cytokines. Under healthy conditions, the production of EC-derived mediators with opposite effects is balanced. However, under pathological conditions, this balance shifts towards vasoconstriction, inflammation, cell proliferation, and thrombosis, turning endothelial cells into propagators of disease [113]. All the classic cardiovascular risk factors cause endothelial dysfunction with a significant reduction in the production or availability of vasodilators, mainly nitric oxide (NO), and a parallel increase in the production of vasoconstrictors, including endothelin-1, angiotensin II, and thromboxane A2 [114].

The function of EC can be indirectly assessed in vivo in humans by stimulating endothelial NO production with pharmacological or mechanical stimuli (endothelial-dependent vasodilation) and comparing the induced vasodilatory response with that induced by an exogenous nitrate donor (endothelial-independent vasodilation) [115]. The difference between endothelial-dependent and endothelial-independent vasodilation is proportional to the extent of endothelial dysfunction [116] which is an independent predictor of cardiovascular events and mortality [117].

Several experimental and human studies have demonstrated that ω-3 PUFAs improve the endothelial response of both normal and damaged endothelium [118]. In EC, incubation with EPA stimulates the production of NO through the activation of endothelial NO synthase (eNOS) [119] leading to endothelial-dependent vasodilation of arterioles [119,120]. NO production by endothelial cells is also indirectly enhanced by ω-3 PUFAs-induced reduction in circulating asymmetric dimethylarginine, a potent endogenous inhibitor of the eNOS activity [121]. Another mechanism contributing to ω-3 PUFA endothelial-dependent regulation of vascular tone is the reduction in oxidative stress [122]. ω-3 PUFAs reduce oxidative stress by decreasing the activity of nicotinamide adenine dinucleotide phosphate [122], blocking the xanthine oxidase pathway [123], and activating the antioxidant enzyme superoxide dismutase (SOD) [124]. 

Some ω-3 oxylipins have also demonstrated antioxidant capacity. Protectin DX derived from DHA, a known isomer of protectin D1, attenuated hydrogen peroxide (H_2_O_2_)-mediated reactive oxygen species production by regulating the expression of antioxidant enzymes including catalase and SOD [83]. Maresin 1/Resolvin D1 also improved SOD and glutathione peroxidase activity and attenuated lipid peroxidation and superoxide anion (O_2_·^−^) generation [864. ω-3 PUFAs showed also regenerative properties over the vascular endothelium. These properties are mediated by stimulation of the endothelial progenitor cells, an effect that has been reported in healthy subjects and patients at high cardiovascular risk [125].

#### 4.2.2. ω-3 PUFAs and Endothelium-Independent Regulation of Vascular Tone

Insight from human and animal studies indicates that, beyond endothelial NO production, there are other mechanisms involved in ω-3 PUFA-mediated regulation of vascular tone. Evidence obtained in studies on blood vessels isolated from experimental animals demonstrates that ω-3 PUFA-mediated vasodilatation occurs even after eNOS inhibition or endothelial removal [126,127,128], suggesting a direct effect on smooth muscle vascular cells (SMVCs). Ca^2+^ homeostasis is the major factor involved in the control of SMVC contraction, which is enhanced by depolarization and resultant Ca^2+^ influx and is decreased by hyperpolarizing K+ efflux. A large body of knowledge exists about the interaction of ω-3 PUFAs with SMVC ion channels, contributing to their vasodilatory properties. These include inhibition of L-type Ca^2+^ channels, activation of K^+^ channels, and activation of TPRV4 (transient receptor potential cation channel subfamily V member 4) channels. Activation of K_ATP_ channels is another attractive potential mechanism for ω-3 PUFA-induced vascular relaxation. Nonetheless, the studies investigating the role of K_ATP_ channels in ω-3 PUFA-mediated vasodilation are limited [129,130,131,132], and the mechanism by which ω-3 PUFA might activate K_ATP_ channels has not yet been clarified [133,134,135]. Thus, further research is still needed to investigate the role of these channels in the regulation of vascular tone and BP and their response to ω-3 PUFAs. 

## 5. ω-3 PUFAs and the Risk of Hypertension Development

As stated above, there is experimental evidence indicating a beneficial effect of ω-3 PUFAs on BP. This is why some epidemiological studies have addressed the possibility that sustained ω-3 PUFA consumption, either as supplements or fishmeal, might reduce the risk of hypertension development. Among the 5394 black and white men and women who were followed for 10 years in the National Health and Nutrition Examination Survey-I (NHANES-I), a significant interaction between regular fish consumption and the development of hypertension (BP ≥ 160/90) was found in black women [136]. The prospective, multicenter Coronary Artery Risk Development in Young Adults (CARDIA) Study [137] examined the association of different foods with the development of hypertension in a multiethnic population. In this study, fish intake, both fresh and processed, was not related to incident hypertension. In the Korean Genome Epidemiology Study, the interaction of fish consumption and ω-3 PUFA supplementation with the development of the metabolic syndrome was investigated over a follow-up of 4 years [138]. After controlling for confounders, the odds ratio for developing the metabolic syndrome in men who ate fish daily in comparison to men who ate fish less than once a week was 0.43. The same significant association was not observed in women. In this study, analysis of the association between fish consumption and separate components of the metabolic syndrome did not show any relationship with the development of hypertension. To examine the association of ω-3 PUFAs with metabolic syndrome in 55 uncomplicated hypertensive patients, we measured the fatty acid composition of red blood cell membranes [139]. Prevalence of the metabolic syndrome was 36% and in patients with metabolic syndrome, the membrane content of ω-3 PUFAs was significantly lower than in patients without the metabolic syndrome.

In the cohort of the Physicians Health Study (PHS), 12.279 normotensive men were followed for 15 years, and the risk of developing hypertension related to fish and ω-3 PUFA consumption was examined [140,141]. Results indicated that incident hypertension was independent of fish and ω-3 PUFA consumption. The incidence of hypertension was not significantly different between men who consumed at least five servings per week of fish compared with those who did not consume any fish and the lack of association extended to individual types of fish. A possible association between fish consumption and the development of hypertension was examined in a meta-analysis of eight observational, prospective studies including 56.204 adults free of cardiovascular disease who were normotensive at baseline and were followed from 3 to 20 years [98]. In this meta-analysis, the risk of developing hypertension was not associated with fish consumption, but the study showed that higher circulating levels of EPA and DHA were significantly associated with a lower risk of incident hypertension. Thus, current data in support of the role of fish or ω-3 PUFA consumption in the prevention of hypertension are extremely weak, whereas lower levels of ω-3 PUFAs are seemingly associated with the presence of the metabolic syndrome.

## 6. ω-3 PUFAs and Hypertension-Related Vascular Damage

### 6.1. Arterial Stiffness

Arterial stiffening is a progressive process that is associated with aging and is accelerated by increased BP and levels of blood lipoproteins that reciprocally interact. Arterial stiffening results from both structural and functional changes to the vascular wall [142]. Aging and additional pathological conditions induce extensive anatomical rearrangement with depletion and fragmentation of elastin fibers and deposition of collagen and matrix metalloproteins, leading to the expansion of the extracellular matrix. On the other hand, endothelial dysfunction with a reduced release of NO contributes to arterial stiffening by increasing the tone of vascular smooth muscle cells. In hypertension, arterial stiffening is an independent predictor of major cardiovascular events. Many noninvasive methods have been used for the assessment of arterial stiffness, and measurement of the carotid-femoral pulse wave velocity (PWV) has become very popular among these methods and is now considered the gold standard. Effects of ω-3 PUFA supplementation on PWV and other indexes of arterial stiffness were analyzed in a systematic review of 10 trials including 550 healthy and hypertensive participants who were randomized to ω-3 PUFA doses ranging from 0.64 to 3.00 g/day or placebo, for a period of 6 to 105 weeks [143]. Treatment with ω-3 PUFAs improved significantly arterial stiffness with an effect that was independent of BP changes. In a randomized, controlled clinical trial on healthy subjects, a high dose of fish oil (6 g/day) improved arterial stiffness more effectively than a low dose (3 g/day) [144].

In addition to PWV, other markers of vascular reactivity such as arterial diameters and endothelium-dependent and independent vascular responses are important predictors of future major cardiovascular events [145,146]. In 45 uncomplicated patients with hypertension, we measured the content of fatty acids in red blood cell membranes as a marker of dietary intake and the vasodilatory response of the brachial artery to both a nitrate donor compound (endothelium-independent vasodilation) and post-ischemic reactive hyperemia (endothelium-dependent flow-mediated dilation) [147]. The baseline caliber of the brachial artery was significantly lower and vasodilatory response to nitrate was significantly greater in patients with higher polyunsaturated-to-saturated fatty acid ratio (PUFA/SFA), whereas no difference was found in flow-mediated dilation. Moreover, PUFA/SFA was independently and inversely related to brachial artery diameter and directly with vasodilatory response to nitrate suggesting a contribution of ω-3 PUFAs to endothelium-independent vascular reactivity. Thus, several lines of evidence strongly suggest that ω-3 PUFAs contribute to vascular reactivity and arterial stiffening.

### 6.2. Atherosclerosis and Plaque Formation

The formation of atherosclerotic plaques is anticipated by the thickening of the inner arterial layer (intima-media thickness, IMT) that can readily be measured by ultrasound. In a prospective intervention study, 56 hypertensive patients received intensive nutritional counseling and three weekly meals of fish containing high amounts of PUFAs and the carotid artery IMT and the red blood cell membrane fatty acid composition with the calculation of the PUFA/SFA ratio were measured at baseline and after 1 year [148]. At baseline, the membrane PUFA/SFA ratio was inversely related to the carotid IMT and at follow-up, PUFA/SFA was increased in 45% of patients. Regular consumption of fish meals resulted in a reduction in carotid IMT only in those patients who had an increased PUFA/SFA ratio that was independent of changes in body mass, BP, and plasma lipids. Very similar conclusions were reported in other studies conducted in different patients’ settings [149,150,151]. ω-3 PUFAs were also proven to stabilize existing atherosclerotic plaques [152] both in critical [152,153] and noncritical carotid artery stenosis [154]. This occurs through the thickening of the plaque’s fibrous cup and reduction in intra-plaque inflammation that are coupled with an increase in HDL-cholesterol [139,155,156] and reduction in lipoprotein(a) [15,157] levels. Finally, it has been demonstrated that ω-3 PUFA supplementation improves the vascular benefits of drugs, such as statins and aspirin [157,158,159].

## 7. Cardiovascular Prevention Studies

Despite several lines of evidence suggesting the existence of multiple beneficial vascular effects of ω-3 PUFAs, results of intervention trials on cardiovascular prevention yielded inconsistent results. This might be due to important differences in subjects involved, types of ω-3 PUFA supplements, and doses and duration of exposure. To our knowledge, no clinical outcome study has included only hypertensive patients, and outcome data of ω-3 PUFA use in hypertension are extrapolated from cardiovascular prevention studies that included substantial proportions of hypertensive patients.

### 7.1. Early Studies

The GISSI (Gruppo Italiano per lo Studio nella Sopravvivenza nell’Infarto Miocardico)-Prevenzione trial included 11,324 patients surviving a recent (less than 3 months) myocardial infarction who were randomly assigned to 1 g/day of ω-3 PUFAs, 300 mg/day vitamin E, both ω-3 PUFA and vitamin D, or related placebo [160]. Hypertensive patients were 36% of the entire study population. Over a 3.5-year follow-up, ω-3 PUFAs lowered significantly (by 10%) the risk of the primary combined cardiovascular endpoint of death, nonfatal myocardial infarction, and stroke. The JELIS (Japan EPA Lipid Intervention Study) was an open-label, primary and secondary prevention trial on 18,645 patients with total cholesterol of 6.5 mmol/L or greater, 35% of whom had hypertension [161]. Patients were randomly assigned to receive either 1800 mg of EPA daily with statin or only statin for 5 years. LDL-cholesterol levels decreased by 25% in both treatment groups, while patients in the EPA group had also a significant reduction in triglyceride levels, and only patients in this group had a significant (by 19%) reduction in the primary composite cardiovascular endpoint. Post-hoc analyses of JELIS showed significant reductions in cardiovascular events with the use of EPA only in secondary prevention settings [162], with the greatest benefit obtained in patients with high baseline plasma triglycerides [163]. The GISSI-Heart Failure study was a randomized, double-blind, placebo-controlled, survival study that enrolled patients with class II–IV heart failure who were randomly assigned to receive ω-3 PUFA 1 g/day or placebo [164]. Hypertensive patients were 55% of the entire study population and patients were followed for a median of 3.9 years. Treatment with ω-3 PUFAs decreased overall and cardiovascular mortality and hospital admissions by 2%, indicating a small but significant benefit. The ORIGIN (Outcome Reduction with an Initial Glargine Intervention) trial was a double-blind study with a 2-by-2 factorial design that randomly assigned 12,536 patients at high risk for cardiovascular events with impaired fasting glucose or diabetes to receive 1 g/day of ω-3 PUFAs (465 mg of EPA and 375 mg of DHA) or placebo and to receive either insulin glargine or standard care [165]. Hypertensive patients were 79% of the studied population, and the primary outcome was death from cardiovascular causes. In this study, no significant benefits of ω-3 PUFAs were observed. The GISSI Risk and Prevention study was a double-blind, placebo-controlled trial, in which 12,513 high-risk patients, 85% of whom had hypertension, were randomly assigned to ω-3 PUFAs 1 g/day or placebo (olive oil) [166]. Patients were followed for a median of 5 years reporting no difference in the primary endpoint that included death, nonfatal myocardial infarction, and nonfatal stroke.

### 7.2. Recent Studies 

Results of early studies could not provide clear-cut conclusions on the potential benefits of ω-3 PUFAs in cardiovascular prevention. This could be possibly related to the widespread use of inadequate doses of ω-3 PUFAs or to the need to have a better selection of patients who could benefit from supplementation. This is why, in recent years, large-scale intervention trials have been performed using greater ω-3 PUFA daily doses and focusing on patients with elevated plasma triglyceride levels. 

The REDUCE-IT (Reduction of Cardiovascular Events with Icosapent Ethyl-Intervention trial) was a randomized, double-blind, placebo-controlled trial involving 8179 patients with established cardiovascular disease or with diabetes, at least one additional cardiovascular risk factor, and plasma triglyceride levels between 135 and 499 mg/dL [23]. Participants were randomized to receive a daily dose of 4 g of icosapent ethyl (IPE, a highly purified EPA preparation) or placebo (mineral oil). After 4.9 years of follow-up, patients treated with IPE had a 25% reduction in the primary endpoint, which was a composite of cardiovascular death, nonfatal myocardial infarction, nonfatal stroke, coronary revascularization, or unstable angina. The results of this study indicated that ω-3 PUFAs could be beneficial in reducing residual cardiovascular risk in patients with high plasma triglycerides, provided that these are used in sufficiently high doses and for the long term. The results of REDUCED-IT prompted the European Medicines Agency (EMA) to include IPE among the strategies recommended for cardiovascular prevention.

ω-3 PUFAs have been tested also in the setting of primary prevention. The STRENGTH (Long-term Outcomes Study to Assess Statin Residual risk with Epanova in High Cardiovascular Risk Patients) study was a double-blind, placebo-controlled trial that recruited 13,078 high cardiovascular risk patients with hypertriglyceridemia, low levels of HDL-cholesterol, and hypertension [167]. Patients received 4 g/day of EPA-DHA or placebo (corn oil) on top of background statin treatment for an average follow-up of 42 months. No difference in the primary composite cardiovascular endpoint was observed between treatment groups. The VITAL (Vitamin D and Omega-3 Trial) was a randomized, placebo-controlled study with a 2-by-2 factorial design of vitamin D and marine ω-3 PUFAs (1 g/day) in the primary prevention of cardiovascular disease and cancer [168]. More than 25,000 participants of both sexes, 49% of whom had hypertension, were followed for 5.3 years during which ω-3 PUFAs did not result in a lower incidence of major cardiovascular events. Another primary prevention study (ASCEND, A Study of Cardiovascular Events in Diabetes) recruited patients with diabetes, 77% of whom had also hypertension [169]. Patients were randomized to receive 1 g/day of ω-3 PUFAs or olive oil and, in a mean follow-up of 7.4 years, the frequency of major cardiovascular events was not different between the two groups.

## 8. Conclusions

Although early studies on the use of ω-3 PUFAs in cardiovascular prevention provided highly controversial results, findings of recent large-scale, randomized clinical trials have reaffirmed the potential advantages of these fatty acids. High-dose EPA formulations have demonstrated significant benefits both in secondary and primary cardiovascular prevention, an effect that has been ascribed to targeting the “residual” cardiovascular risk linked to triglyceride-rich lipoproteins. However, the benefits of ω-3 PUFAs might go beyond triglyceride-lowering, encompassing a broad range of additional “pleiotropic” actions. These include protection from vascular inflammation and thrombosis, improvement of endothelial function, and reduction in BP. In vitro and in vivo animal and human studies suggest that ω-3 PUFAs could improve peripheral resistance to blood flow by affecting both endothelium-dependent and endothelium-independent vascular responses. These vascular responses to ω-3 PUFAs might translate into BP reduction and evidence has been obtained across multiple trials, systematic reviews, and meta-analyses. In these studies, daily intake of 3 or more g/day of ω-3 PUFAs caused significant BP reduction, particularly in subjects with untreated hypertension. Moreover, by decreasing arterial stiffening, slowing the development of atherosclerosis, and increasing arterial plaque stability, supplementation with ω-3 PUFAs intervenes favorably and at different stages of hypertension-related arterial damage.

Despite these intriguing observations, the available evidence of the benefits of ω-3 PUFAs in hypertension has never been considered worthy of specific recommendations to be included in hypertension guidelines [1,2]. This is because no cardiovascular prevention study with the use of ω-3 PUFAs has ever been specifically performed in hypertensive patients. This is why outcome data of ω-3 PUFA’s use in hypertension have been extrapolated from other studies that included subsets of hypertensive patients. While waiting for the necessary evidence, we suggest that high-dose and long-term ω-3 PUFAs supplementation might be considered in (a) low-risk patients with grade 1 hypertension who are unwilling to start drug treatment and will implement all interventions on lifestyle correction recommended by the guidelines; (b) patients with hypertension who meet the inclusion criteria of the REDUCE-IT trial (patients older than 45 years with established cardiovascular disease or older than 50 years with diabetes and one or more major cardiovascular risk factor presenting with a serum triglyceride level of 135 mg/dL or more) [23].

## Figures and Tables

**Table 1:** Pathophysiological mechanisms potentially involved in the antihypertensive action of omega-3 polyunsaturated fatty acids.

Cardiac Hemodynamics	Endothelium-Dependent Regulation of Vascular Tone	Endothelium-Independent Regulation of Vascular Tone
Slowing of heart rate	Increased nitric oxide generation	Inhibition of L-type Ca^2+^ channels
Increased stroke volume	Decreased oxidative stress	Activation of K^+^ channels
	Increased regeneration of endothelial cells	Activation of TPRV4 channel
		Inhibition of TRPC1/5 channels

TRPV-4, transient receptor potential vanilloid-4; TRPC1/5, transient receptor potential cation.

## Data Availability

Not applicable.
